# Na_4.25_Mo_15_S_19_: a novel ternary reduced molybdenum sulfide containing Mo_6_ and Mo_9_ clusters

**DOI:** 10.1107/S160053681401201X

**Published:** 2014-05-31

**Authors:** D. Salloum, P. Gougeon, P. Gall

**Affiliations:** a Faculty of Science III, Lebanese University, PO Box 826, Kobbeh–Tripoli, Lebanon; b Unité Sciences Chimiques de Rennes, UMR CNRS No. 6226, Université de Rennes I – INSA Rennes, Campus de Beaulieu, 35042 Rennes Cedex, France

## Abstract

The structure of tetra­sodium penta­deca­molybdenum nona­deca­sulfide, Na_4.25_Mo_15_S_19_, is isotypic with Na_3.9_Mo_15_Se_19_ [Salloum *et al.* (2013 ▶). *Acta Cryst.* E**69**, i67–i68]. It is characterized by Mo_6_S^*i*^_8_S^*a*^_6_ and Mo_9_S^*i*^_11_S^*a*^_6_ (where *i* represents inner and *a* apical atoms) cluster units that are present in a 1:1 ratio. The cluster units are centered at Wyckoff positions 2*b* and 2*c*, and have point-group symmetry -3 and -6, respectively. The clusters are inter­connected through additional Mo—S bonds. The Na^+^ cations occupy inter­unit voids formed by six or seven S atoms. One Mo, one S and one Na site [occupancy 0.751 (12)] are situated on mirror planes, and two other S atoms and one Na site (full occupancy) are situated on threefold rotation axes.

## Related literature   

For previous reports on the crystal structure of the In_∼3_Mo_15_Se_19_ compounds, see: Grüttner *et al.* (1979 ▶). For physical properties of this type of compounds, see: Seeber *et al.* (1979 ▶). The crystal structures of the substituted selenides Ho_0.76_In_1.68_Mo_15_Se_19_ and In_0.87_K_2_Mo_15_Se_19_ were reported by Salloum *et al.* (2006 ▶; 2007 ▶). For the isotypic sulfides In_3.7_Mo_15_S_19_, In_1.6_Rb_2_Mo_15_S_19_, In_2.2_CsMo_15_S_19_, ScTl_2_Mo_15_S_19_ and Na_3.9_Mo_15_Se_19_, see: Salloum *et al.* (2004*a* ▶,*b* ▶, 2013 ▶) and for V_1.42_In_1.83_Mo_15_Se_19_, see: Gougeon *et al.* (2010 ▶). For details of the *i*- and *a*-type ligand notation, see: Schäfer & von Schnering (1964 ▶).

## Experimental   

### 

#### Crystal data  

Na_4.25_Mo_15_S_19_
*M* *_r_* = 2145.95
Hexagonal, 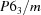

*a* = 9.5340 (1) Å
*c* = 18.9803 (3) Å
*V* = 1494.11 (3) Å^3^
*Z* = 2
Mo *K*α radiation
μ = 7.44 mm^−1^
*T* = 293 K
0.18 × 0.14 × 0.08 mm

#### Data collection  

Nonius KappaCCD diffractometer
Absorption correction: analytical (de Meulenaar & Tompa, 1965 ▶) *T*_min_ = 0.363, *T*_max_ = 0.591
16576 measured reflections
1500 independent reflections
1322 reflections with *I* > 2σ(*I*)
*R*_int_ = 0.085

#### Refinement  

*R*[*F* ^2^ > 2σ(*F* ^2^)] = 0.030
*wR*(*F* ^2^) = 0.079
*S* = 1.13
1500 reflections
66 parameters
Δρ_max_ = 1.51 e Å^−3^
Δρ_min_ = −1.85 e Å^−3^

### 

Data collection: *COLLECT* (Nonius, 1998 ▶); cell refinement: *COLLECT*; data reduction: *EVALCCD* (Duisenberg, 1998 ▶); program(s) used to solve structure: *SIR97* (Altomare *et al.*, 1999 ▶); program(s) used to refine structure: *SHELXL97* (Sheldrick, 2008 ▶); molecular graphics: *DIAMOND* (Bergerhoff, 1996 ▶); software used to prepare material for publication: *SHELXL97*.

## Supplementary Material

Crystal structure: contains datablock(s) I, global. DOI: 10.1107/S160053681401201X/ru2060sup1.cif

Structure factors: contains datablock(s) I. DOI: 10.1107/S160053681401201X/ru2060Isup2.hkl

CCDC reference: 1004866

Additional supporting information:  crystallographic information; 3D view; checkCIF report

## Figures and Tables

**Fig. 1.**
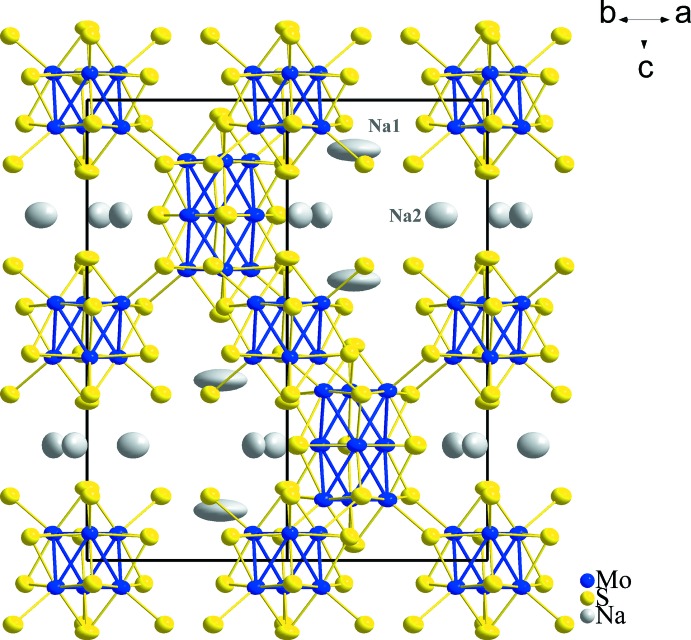
: View of Na4.25Mo15S19 along [110]. Displacement ellipsoids are drawn at the 97% probability level.

**Fig. 2.**
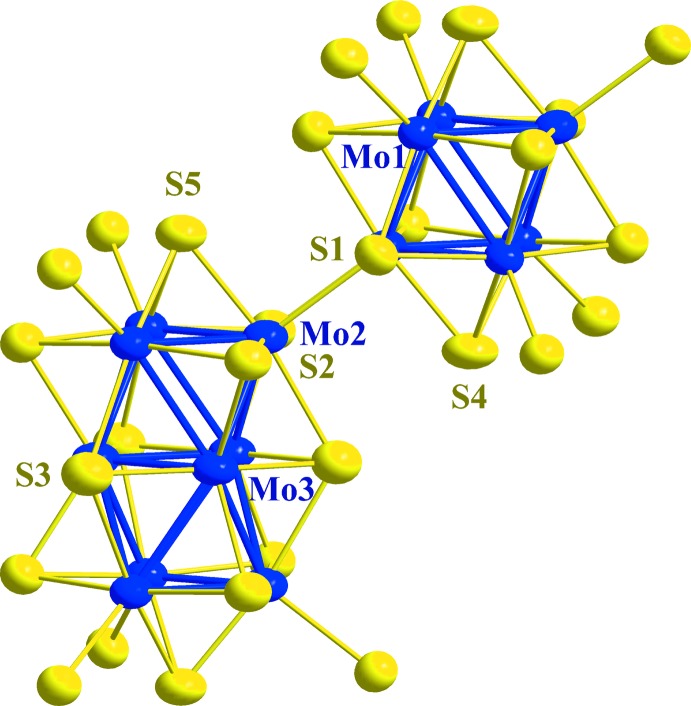
Plot showing the atom-numbering scheme and the interunit linkage of the Mo9S11S6 and Mo6S8S6 cluster units. Displacement ellipsoids are drawn at the 97% probability level.

## supplementary crystallographic information

### 1. Comment

The reduced molybdenum compounds In_3 +_*_x_*Mo_15_*X*_19_ (*X* = S, Se) (Grüttner *et al.*, 1979; Salloum *et al.*, 2004*a*) crystallize in an interesting structural type characterized by an equal mixture of Mo_6_ and Mo_9_ clusters and by In atoms that occupy two or three different crystallographically positions depending on their formal oxidation state of +1 or +3. Subsequently, isomorphous compounds such as Ho_0.76_In_1.68_Mo_15_Se_19_ (Salloum *et al.*, 2006), In_0.87_K_2_Mo_15_Se_19_ (Salloum *et al.*, 2007), V_1.42_In_1.83_Mo_15_Se_19_ (Gougeon *et al.*, 2010), In_3.7_Mo_15_S_19_ (Salloum *et al.*, 2004*a*), In_1.6_Rb_2_Mo_15_S_19_, In_2.2_CsMo_15_S_19_ and ScTl_2_Mo_15_S_19_ (Salloum *et al.*, 2004*b*) have been synthesized. In the latter compounds, the Ho, V and Sc atoms replace the trivalent indium and the K, Cs, and Tl atoms the monovalent one. Recently, we described the crystal structure of Na_3.9_Mo_15_Se_19_ (Salloum *et al.*, 2013) in which the sodium replaces the monovalent as well as the trivalent indium for the first time. We present here the sulfide analogue Na_4.25_Mo_15_S_19_.

The Mo—S framework of the title compound consists of the cluster units Mo_6_S^i^_8_S*^a^*_6_ and Mo_9_S^i^_11_S*^a^*_6_ in a 1:1 ratio (for details of the i- and a-type ligand notation, see Schäfer & von Schnering (1964)). Both components are interconnected through additional Mo—Se bonds (Figs. 1 and 2). The first unit can be described as an Mo_6_ octahedron surrounded by eight face-capping inner S^i^ and six apical S*^a^* ligands. The Mo_9_ cluster is surrounded by 11 S^i^ atoms capping one or two faces of the bioctahedron and six S*^a^* ligands above the apical Mo atoms. The Mo_6_S^i^_8_S*^a^*_6_ and Mo_9_S^i^_11_S*^a^*_6_ units are centered at Wyckoff positions 2 b and 2c and have point-group symmetry 3 and 6, respectively. The Mo—Mo distances within the Mo_6_ cluster are 2.6900 (5) Å for the distances of the Mo triangles formed by the Mo1 atoms related through the threefold axis, and 2.7098 (6) Å for the distances between these triangles. The Mo—Mo distances within the Mo_9_ clusters are 2.6349 (5) and 2.6756 (7) Å in the triangles formed by the atoms Mo2 and Mo3, respectively, and 2.7081 (4) and 2.7303 (4) Å for those between the Mo2_3_ and Mo3_3_ triangles. All the latter Mo—Mo distances are closed to those observed in the selenide analogue indicating that the cationic charge transfer towards the Mo_6_ and Mo_9_ clusters are similar in both compounds. The S atoms bridge either one (S1, S2, S4 and S5) or two (S3) triangular faces of the Mo clusters. Moreover, atoms S1 and S2 are linked to an Mo atom of a neighboring cluster. The Mo—S bond distances range from 2.4184 (14) to 2.5624 (10) Å within the Mo_6_S^i^_8_S*^a^*_6_ unit, and from 2.4033 (13) to 2.5947 (8) Å within the Mo_9_S^i^_11_S*^a^*_6_ unit. In both cases, the shortest bonds involve the S4 and S5 terminal atoms and the longest ones correspond to the interunit Mo1—S2 and Mo2—S1 bonds. Each Mo_9_S^i^_11_S*^a^*_6_ cluster is thus interconnected to six Mo_6_S^i^_8_S*^a^*_6_ units (and *vice versa*) *via* Mo2—S1 bonds (and Mo1—S2 bonds, respectively), forming the three-dimensional Mo—S framework, the connective formula of which is Mo_9_S^i^_5_S^i-a^_6/2_S^a-i^_6/2_, Mo_6_S^i^_2_S^i-a^_6/2_S^a-i^_6/2_. It results from this arrangement that the shortest intercluster Mo1—Mo2 distance is 3.5202 (6) Å, indicating only weak metal-metal interactions between the Mo clusters. The Na1^+^ cations are surrounded by seven S atoms forming a distorted tricapped tetrahedron. The S5 and S2 atoms forming the tetrahedron are at 2.699 (5) and 3.1669 (13) Å from the Na1 atom, and the capping S1 atoms are at 3.3609 (19) Å. The Na2^+^ cations occupy partially at 75.1% a triangular group of distorted octahedral cavities around the threefold axis, which are formed by two Mo_6_S^i^_8_S*^a^*_6_ and three Mo_9_S^i^_11_S*^a^*_6_ units. The Na2—S distances are in the 2.538 (4) - 3.055 (4) Å range.

### 2. Experimental

Single crystals of Na_4.25_Mo_15_S_19_ were prepared from an ion exchange reaction on single crystals of In_3 +_*_x_*Mo_15_S_19_ with an excess of NaI at 1073 K. The mixture was sealed under vacuum in a long silica tube. The end of tube containing the crystals of In_3 +_*_x_*Mo_15_S_19_ and InI was placed in a furnace with about 5 cm of the other end out from the furnace, at about the room temperature. The furnace was heated at 1073 K for 48 h. After reaction, crystals of InI were observed at the cool end of the tube. The black crystals of the title compound were subsequently washed with water to remove the excess of InI. Qualitative microanalyses using a Jeol JSM 6400 scanning electron microscope equipped with a Oxford INCA energy- dispersive-type X-ray spectrometer did not reveal the presence of indium in the crystals and indicated roughly stoichiometries comprised between 3.8 and 4.4 for the Na content.

### 3. Refinement

No significant deviation from full occupancy was observed for Na1. The site occupation factor of Na2 was refined freely leading to the final stoichiometry Na_4.25 (4)_Mo_15_S_19_.

### Crystal data

**Table:**

Na_4.25_Mo_15_S_19_	*D*_x_ = 4.770 Mg m^−^^3^
*M**_r_* = 2145.95	Mo *K*α radiation, λ = 0.71069 Å
Hexagonal, *P*6_3_/*m*	Cell parameters from 16576 reflections
*a* = 9.5340 (1) Å	θ = 2.2–30.0°
*c* = 18.9803 (3) Å	µ = 7.44 mm^−^^1^
*V* = 1494.11 (3) Å^3^	*T* = 293 K
*Z* = 2	Multi-faceted crystal, black
*F*(000) = 1962	0.18 × 0.14 × 0.08 mm

### Data collection

**Table:**

Nonius KappaCCD diffractometer	1500 independent reflections
Radiation source: fine-focus sealed tube	1322 reflections with *I* > 2σ(*I*)
Graphite monochromator	*R*_int_ = 0.085
φ scans (κ = 0) + additional ω scans	θ_max_ = 30.0°, θ_min_ = 2.2°
Absorption correction: analytical (de Meulenaar & Tompa, 1965)	*h* = −12→13
*T*_min_ = 0.363, *T*_max_ = 0.591	*k* = −13→11
16576 measured reflections	*l* = −22→26

### Refinement

**Table:**

Refinement on *F*^2^	Primary atom site location: structure-invariant direct methods
Least-squares matrix: full	Secondary atom site location: difference Fourier map
*R*[*F*^2^ > 2σ(*F*^2^)] = 0.030	*w* = 1/[σ^2^(*F*_o_^2^) + (0.0337*P*)^2^ + 5.1576*P*] where *P* = (*F*_o_^2^ + 2*F*_c_^2^)/3
*wR*(*F*^2^) = 0.079	(Δ/σ)_max_ = 0.001
*S* = 1.13	Δρ_max_ = 1.51 e Å^−^^3^
1500 reflections	Δρ_min_ = −1.85 e Å^−^^3^
66 parameters	Extinction correction: *SHELXL97* (Sheldrick, 2008), Fc^*^=kFc[1+0.001xFc^2^λ^3^/sin(2θ)]^-1/4^
0 restraints	Extinction coefficient: 0.00266 (19)

### Special details

**Table:**

Geometry. All e.s.d.'s (except the e.s.d. in the dihedral angle between two l.s. planes) are estimated using the full covariance matrix. The cell e.s.d.'s are taken into account individually in the estimation of e.s.d.'s in distances, angles and torsion angles; correlations between e.s.d.'s in cell parameters are only used when they are defined by crystal symmetry. An approximate (isotropic) treatment of cell e.s.d.'s is used for estimating e.s.d.'s involving l.s. planes.
Refinement. Refinement of *F*^2^ against ALL reflections. The weighted *R*-factor *wR* and goodness of fit *S* are based on *F*^2^, conventional *R*-factors *R* are based on *F*, with *F* set to zero for negative *F*^2^. The threshold expression of *F*^2^ > σ(*F*^2^) is used only for calculating *R*-factors(gt) *etc*. and is not relevant to the choice of reflections for refinement. *R*-factors based on *F*^2^ are statistically about twice as large as those based on *F*, and *R*- factors based on ALL data will be even larger.

### Fractional atomic coordinates and isotropic or equivalent isotropic displacement parameters (Å^2^)

**Table:**

	*x*	*y*	*z*	*U*_iso_*/*U*_eq_	Occ. (<1)
Mo1	0.84424 (3)	0.01344 (3)	−0.058496 (18)	0.01072 (12)	
Mo2	0.50077 (4)	−0.18303 (4)	0.131678 (19)	0.01177 (12)	
Mo3	0.34797 (5)	−0.16448 (5)	0.2500	0.01301 (13)	
S1	0.71650 (10)	0.02755 (11)	0.05118 (5)	0.01268 (18)	
S2	0.36949 (11)	−0.01601 (11)	0.13948 (5)	0.01344 (19)	
S3	0.05126 (16)	−0.30754 (17)	0.2500	0.0171 (3)	
S4	0.0000	0.0000	−0.15617 (9)	0.0184 (3)	
S5	0.3333	−0.3333	0.03365 (9)	0.0156 (3)	
Na2	0.7703 (5)	−0.0623 (4)	−0.2500	0.0283 (12)	0.751 (12)
Na1	0.3333	−0.3333	−0.1085 (3)	0.0789 (17)	

### Atomic displacement parameters (Å^2^)

**Table:**

	*U*^11^	*U*^22^	*U*^33^	*U*^12^	*U*^13^	*U*^23^
Mo1	0.01252 (16)	0.01051 (16)	0.00816 (19)	0.00505 (11)	0.00061 (10)	−0.00031 (9)
Mo2	0.01434 (17)	0.01410 (17)	0.00778 (19)	0.00778 (12)	−0.00017 (10)	−0.00005 (10)
Mo3	0.0153 (2)	0.0159 (2)	0.0075 (2)	0.00754 (17)	0.000	0.000
S1	0.0121 (4)	0.0138 (4)	0.0121 (4)	0.0064 (3)	0.0013 (3)	0.0005 (3)
S2	0.0154 (4)	0.0143 (4)	0.0112 (4)	0.0079 (3)	0.0001 (3)	0.0008 (3)
S3	0.0185 (6)	0.0198 (6)	0.0133 (7)	0.0098 (5)	0.000	0.000
S4	0.0228 (5)	0.0228 (5)	0.0095 (8)	0.0114 (2)	0.000	0.000
S5	0.0187 (4)	0.0187 (4)	0.0093 (7)	0.0094 (2)	0.000	0.000
Na2	0.040 (2)	0.0256 (19)	0.026 (2)	0.0218 (16)	0.000	0.000
Na1	0.109 (3)	0.109 (3)	0.018 (2)	0.0547 (14)	0.000	0.000

### Geometric parameters (Å, º)

**Table:**

Mo1—S4^i^	2.4184 (14)	S2—Mo1^iv^	2.5624 (10)
Mo1—S1	2.4492 (10)	S2—Na2^iv^	2.781 (2)
Mo1—S1^ii^	2.4565 (9)	S2—Na1^iv^	3.1669 (13)
Mo1—S1^iii^	2.4830 (9)	S3—Mo3^viii^	2.4419 (14)
Mo1—S2^iv^	2.5624 (10)	S3—Na2^xi^	2.538 (4)
Mo1—Mo1^v^	2.6900 (5)	S3—Mo2^x^	2.5947 (8)
Mo1—Mo1^vi^	2.6900 (5)	S3—Mo2^viii^	2.5947 (8)
Mo1—Mo1^iii^	2.7098 (6)	S3—Na2^iv^	3.055 (4)
Mo1—Mo1^ii^	2.7098 (6)	S4—Mo1^xii^	2.4184 (14)
Mo1—Na2	3.7042 (8)	S4—Mo1^xiii^	2.4184 (14)
Mo2—S5	2.4033 (13)	S4—Mo1^viii^	2.4184 (14)
Mo2—S2	2.4733 (9)	S4—Na2^xiii^	2.649 (3)
Mo2—S2^vii^	2.5070 (9)	S4—Na2^viii^	2.649 (3)
Mo2—S1	2.5429 (10)	S4—Na2^xii^	2.649 (3)
Mo2—S3^vii^	2.5947 (8)	S5—Mo2^viii^	2.4033 (13)
Mo2—Mo2^vii^	2.6349 (5)	S5—Mo2^vii^	2.4033 (13)
Mo2—Mo2^viii^	2.6349 (5)	S5—Na1	2.699 (5)
Mo2—Mo3^vii^	2.7081 (4)	Na2—S3^xiv^	2.538 (4)
Mo2—Mo3	2.7303 (4)	Na2—S4^i^	2.649 (3)
Mo3—S3^vii^	2.4419 (14)	Na2—S4^xv^	2.649 (3)
Mo3—S3	2.4504 (14)	Na2—S2^xvi^	2.781 (2)
Mo3—S2^ix^	2.4810 (10)	Na2—S2^iv^	2.781 (2)
Mo3—S2	2.4810 (10)	Na2—Mo3^iv^	2.896 (3)
Mo3—Mo3^viii^	2.6756 (7)	Na2—S3^iv^	3.055 (4)
Mo3—Mo3^vii^	2.6756 (7)	Na2—Na2^v^	3.397 (6)
Mo3—Mo2^viii^	2.7081 (4)	Na2—Na2^vi^	3.397 (6)
Mo3—Mo2^x^	2.7081 (4)	Na2—Mo1^xvii^	3.7042 (8)
Mo3—Mo2^ix^	2.7303 (4)	Na1—S2^xviii^	3.1669 (13)
Mo3—Na2^iv^	2.896 (3)	Na1—S2^iv^	3.1669 (13)
S1—Mo1^iii^	2.4565 (9)	Na1—S2^ii^	3.1669 (13)
S1—Mo1^ii^	2.4830 (9)	Na1—S1^iv^	3.3609 (19)
S1—Na1^iv^	3.3609 (19)	Na1—S1^xviii^	3.3609 (19)
S2—Mo2^viii^	2.5070 (9)	Na1—S1^ii^	3.3609 (19)
			
S4^i^—Mo1—S1	171.81 (4)	Mo1—S1—Mo1^ii^	66.65 (3)
S4^i^—Mo1—S1^ii^	90.83 (2)	Mo1^iii^—S1—Mo1^ii^	65.99 (3)
S1—Mo1—S1^ii^	89.17 (2)	Mo1—S1—Mo2	134.06 (4)
S4^i^—Mo1—S1^iii^	90.20 (2)	Mo1^iii^—S1—Mo2	130.92 (4)
S1—Mo1—S1^iii^	88.56 (2)	Mo1^ii^—S1—Mo2	82.94 (3)
S1^ii^—Mo1—S1^iii^	171.12 (4)	Mo1—S1—Na1^iv^	127.79 (8)
S4^i^—Mo1—S2^iv^	93.04 (4)	Mo1^iii^—S1—Na1^iv^	97.39 (3)
S1—Mo1—S2^iv^	95.15 (3)	Mo1^ii^—S1—Na1^iv^	153.45 (7)
S1^ii^—Mo1—S2^iv^	91.20 (3)	Mo2—S1—Na1^iv^	94.64 (6)
S1^iii^—Mo1—S2^iv^	97.55 (3)	Mo2—S2—Mo3	66.88 (3)
S4^i^—Mo1—Mo1^v^	56.21 (2)	Mo2—S2—Mo2^viii^	63.88 (2)
S1—Mo1—Mo1^v^	116.82 (2)	Mo3—S2—Mo2^viii^	65.76 (2)
S1^ii^—Mo1—Mo1^v^	117.37 (2)	Mo2—S2—Mo1^iv^	129.13 (4)
S1^iii^—Mo1—Mo1^v^	56.53 (2)	Mo3—S2—Mo1^iv^	132.33 (4)
S2^iv^—Mo1—Mo1^v^	135.73 (2)	Mo2^viii^—S2—Mo1^iv^	82.07 (3)
S4^i^—Mo1—Mo1^vi^	56.21 (2)	Mo2—S2—Na2^iv^	133.09 (7)
S1—Mo1—Mo1^vi^	117.46 (2)	Mo3—S2—Na2^iv^	66.52 (6)
S1^ii^—Mo1—Mo1^vi^	57.48 (2)	Mo2^viii^—S2—Na2^iv^	100.94 (8)
S1^iii^—Mo1—Mo1^vi^	116.43 (2)	Mo1^iv^—S2—Na2^iv^	87.68 (6)
S2^iv^—Mo1—Mo1^vi^	131.85 (2)	Mo2—S2—Na1^iv^	101.03 (3)
Mo1^v^—Mo1—Mo1^vi^	60.0	Mo3—S2—Na1^iv^	122.15 (9)
S4^i^—Mo1—Mo1^iii^	116.37 (2)	Mo2^viii^—S2—Na1^iv^	160.14 (7)
S1—Mo1—Mo1^iii^	56.60 (2)	Mo1^iv^—S2—Na1^iv^	100.15 (7)
S1^ii^—Mo1—Mo1^iii^	115.84 (3)	Na2^iv^—S2—Na1^iv^	98.88 (10)
S1^iii^—Mo1—Mo1^iii^	56.08 (2)	Mo3^viii^—S3—Mo3	66.31 (4)
S2^iv^—Mo1—Mo1^iii^	138.14 (2)	Mo3^viii^—S3—Na2^xi^	157.19 (11)
Mo1^v^—Mo1—Mo1^iii^	60.241 (8)	Mo3—S3—Na2^xi^	136.50 (11)
Mo1^vi^—Mo1—Mo1^iii^	90.0	Mo3^viii^—S3—Mo2^x^	65.57 (3)
S4^i^—Mo1—Mo1^ii^	116.37 (2)	Mo3—S3—Mo2^x^	64.86 (3)
S1—Mo1—Mo1^ii^	57.27 (2)	Na2^xi^—S3—Mo2^x^	119.39 (3)
S1^ii^—Mo1—Mo1^ii^	56.34 (2)	Mo3^viii^—S3—Mo2^viii^	65.57 (3)
S1^iii^—Mo1—Mo1^ii^	115.54 (3)	Mo3—S3—Mo2^viii^	64.86 (3)
S2^iv^—Mo1—Mo1^ii^	134.12 (2)	Na2^xi^—S3—Mo2^viii^	119.39 (3)
Mo1^v^—Mo1—Mo1^ii^	90.0	Mo2^x^—S3—Mo2^viii^	119.89 (5)
Mo1^vi^—Mo1—Mo1^ii^	60.241 (8)	Mo3^viii^—S3—Na2^iv^	128.66 (8)
Mo1^iii^—Mo1—Mo1^ii^	59.518 (16)	Mo3—S3—Na2^iv^	62.35 (7)
S4^i^—Mo1—Na2	45.53 (6)	Na2^xi^—S3—Na2^iv^	74.15 (16)
S1—Mo1—Na2	142.51 (6)	Mo2^x^—S3—Na2^iv^	92.16 (5)
S1^ii^—Mo1—Na2	83.36 (6)	Mo2^viii^—S3—Na2^iv^	92.16 (5)
S1^iii^—Mo1—Na2	103.55 (6)	Mo1^xii^—S4—Mo1^xiii^	67.58 (5)
S2^iv^—Mo1—Na2	48.59 (6)	Mo1^xii^—S4—Mo1^viii^	67.58 (5)
Mo1^v^—Mo1—Na2	99.04 (6)	Mo1^xiii^—S4—Mo1^viii^	67.58 (5)
Mo1^vi^—Mo1—Na2	88.97 (5)	Mo1^xii^—S4—Na2^xiii^	151.15 (7)
Mo1^iii^—Mo1—Na2	156.07 (6)	Mo1^xiii^—S4—Na2^xiii^	93.82 (5)
Mo1^ii^—Mo1—Na2	137.75 (5)	Mo1^viii^—S4—Na2^xiii^	127.12 (7)
S5—Mo2—S2	91.79 (2)	Mo1^xii^—S4—Na2^viii^	127.12 (7)
S5—Mo2—S2^vii^	90.96 (2)	Mo1^xiii^—S4—Na2^viii^	151.15 (7)
S2—Mo2—S2^vii^	172.09 (4)	Mo1^viii^—S4—Na2^viii^	93.82 (5)
S5—Mo2—S1	92.26 (3)	Na2^xiii^—S4—Na2^viii^	79.75 (9)
S2—Mo2—S1	89.86 (3)	Mo1^xii^—S4—Na2^xii^	93.82 (5)
S2^vii^—Mo2—S1	97.44 (3)	Mo1^xiii^—S4—Na2^xii^	127.12 (7)
S5—Mo2—S3^vii^	170.70 (4)	Mo1^viii^—S4—Na2^xii^	151.15 (7)
S2—Mo2—S3^vii^	86.62 (4)	Na2^xiii^—S4—Na2^xii^	79.75 (9)
S2^vii^—Mo2—S3^vii^	89.47 (4)	Na2^viii^—S4—Na2^xii^	79.75 (9)
S1—Mo2—S3^vii^	96.89 (3)	Mo2^viii^—S5—Mo2	66.48 (4)
S5—Mo2—Mo2^vii^	56.76 (2)	Mo2^viii^—S5—Mo2^vii^	66.48 (4)
S2—Mo2—Mo2^vii^	118.56 (2)	Mo2—S5—Mo2^vii^	66.48 (4)
S2^vii^—Mo2—Mo2^vii^	57.44 (2)	Mo2^viii^—S5—Na1	140.73 (3)
S1—Mo2—Mo2^vii^	136.00 (2)	Mo2—S5—Na1	140.73 (3)
S3^vii^—Mo2—Mo2^vii^	116.29 (3)	Mo2^vii^—S5—Na1	140.73 (3)
S5—Mo2—Mo2^viii^	56.76 (2)	S3^xiv^—Na2—S4^i^	89.92 (9)
S2—Mo2—Mo2^viii^	58.68 (2)	S3^xiv^—Na2—S4^xv^	89.92 (9)
S2^vii^—Mo2—Mo2^viii^	117.32 (2)	S4^i^—Na2—S4^xv^	84.49 (12)
S1—Mo2—Mo2^viii^	131.42 (2)	S3^xiv^—Na2—S2^xvi^	112.74 (10)
S3^vii^—Mo2—Mo2^viii^	115.08 (3)	S4^i^—Na2—S2^xvi^	154.21 (15)
Mo2^vii^—Mo2—Mo2^viii^	60.0	S4^xv^—Na2—S2^xvi^	83.46 (4)
S5—Mo2—Mo3^vii^	118.14 (2)	S3^xiv^—Na2—S2^iv^	112.74 (10)
S2—Mo2—Mo3^vii^	115.63 (3)	S4^i^—Na2—S2^iv^	83.46 (4)
S2^vii^—Mo2—Mo3^vii^	56.66 (2)	S4^xv^—Na2—S2^iv^	154.21 (15)
S1—Mo2—Mo3^vii^	137.83 (3)	S2^xvi^—Na2—S2^iv^	97.94 (11)
S3^vii^—Mo2—Mo3^vii^	54.99 (3)	S3^xiv^—Na2—Mo3^iv^	145.61 (16)
Mo2^vii^—Mo2—Mo3^vii^	61.444 (11)	S4^i^—Na2—Mo3^iv^	114.79 (9)
Mo2^viii^—Mo2—Mo3^vii^	90.670 (10)	S4^xv^—Na2—Mo3^iv^	114.79 (9)
S5—Mo2—Mo3	117.29 (2)	S2^xvi^—Na2—Mo3^iv^	51.78 (6)
S2—Mo2—Mo3	56.69 (2)	S2^iv^—Na2—Mo3^iv^	51.78 (6)
S2^vii^—Mo2—Mo3	115.55 (3)	S3^xiv^—Na2—S3^iv^	165.85 (15)
S1—Mo2—Mo3	133.65 (3)	S4^i^—Na2—S3^iv^	79.65 (9)
S3^vii^—Mo2—Mo3	54.52 (3)	S4^xv^—Na2—S3^iv^	79.65 (9)
Mo2^vii^—Mo2—Mo3	90.184 (10)	S2^xvi^—Na2—S3^iv^	75.81 (8)
Mo2^viii^—Mo2—Mo3	60.600 (11)	S2^iv^—Na2—S3^iv^	75.81 (8)
Mo3^vii^—Mo2—Mo3	58.940 (16)	Mo3^iv^—Na2—S3^iv^	48.54 (6)
S3^vii^—Mo3—S3	173.69 (4)	S3^xiv^—Na2—Na2^v^	119.90 (13)
S3^vii^—Mo3—S2^ix^	89.88 (3)	S4^i^—Na2—Na2^v^	50.13 (5)
S3—Mo3—S2^ix^	93.48 (3)	S4^xv^—Na2—Na2^v^	50.13 (5)
S3^vii^—Mo3—S2	89.88 (3)	S2^xvi^—Na2—Na2^v^	105.50 (12)
S3—Mo3—S2	93.48 (3)	S2^iv^—Na2—Na2^v^	105.50 (12)
S2^ix^—Mo3—S2	115.45 (4)	Mo3^iv^—Na2—Na2^v^	94.49 (15)
S3^vii^—Mo3—Mo3^viii^	117.00 (4)	S3^iv^—Na2—Na2^v^	45.95 (11)
S3—Mo3—Mo3^viii^	56.69 (4)	S3^xiv^—Na2—Na2^vi^	59.90 (13)
S2^ix^—Mo3—Mo3^viii^	118.47 (2)	S4^i^—Na2—Na2^vi^	50.13 (5)
S2—Mo3—Mo3^viii^	118.47 (2)	S4^xv^—Na2—Na2^vi^	50.13 (5)
S3^vii^—Mo3—Mo3^vii^	57.00 (4)	S2^xvi^—Na2—Na2^vi^	130.76 (6)
S3—Mo3—Mo3^vii^	116.69 (4)	S2^iv^—Na2—Na2^vi^	130.76 (6)
S2^ix^—Mo3—Mo3^vii^	116.54 (2)	Mo3^iv^—Na2—Na2^vi^	154.49 (15)
S2—Mo3—Mo3^vii^	116.54 (2)	S3^iv^—Na2—Na2^vi^	105.95 (11)
Mo3^viii^—Mo3—Mo3^vii^	60.0	Na2^v^—Na2—Na2^vi^	60.0
S3^vii^—Mo3—Mo2^viii^	117.827 (15)	S3^xiv^—Na2—Mo1	97.96 (5)
S3—Mo3—Mo2^viii^	60.149 (14)	S4^i^—Na2—Mo1	40.65 (3)
S2^ix^—Mo3—Mo2^viii^	150.01 (3)	S4^xv^—Na2—Mo1	124.10 (12)
S2—Mo3—Mo2^viii^	57.58 (2)	S2^xvi^—Na2—Mo1	139.27 (11)
Mo3^viii^—Mo3—Mo2^viii^	60.944 (12)	S2^iv^—Na2—Mo1	43.72 (2)
Mo3^vii^—Mo3—Mo2^viii^	89.804 (10)	Mo3^iv^—Na2—Mo1	87.79 (5)
S3^vii^—Mo3—Mo2^x^	117.827 (15)	S3^iv^—Na2—Mo1	80.39 (5)
S3—Mo3—Mo2^x^	60.149 (14)	Na2^v^—Na2—Mo1	79.33 (6)
S2^ix^—Mo3—Mo2^x^	57.58 (2)	Na2^vi^—Na2—Mo1	87.34 (5)
S2—Mo3—Mo2^x^	150.01 (3)	S3^xiv^—Na2—Mo1^xvii^	97.96 (5)
Mo3^viii^—Mo3—Mo2^x^	60.944 (12)	S4^i^—Na2—Mo1^xvii^	124.10 (12)
Mo3^vii^—Mo3—Mo2^x^	89.804 (10)	S4^xv^—Na2—Mo1^xvii^	40.65 (3)
Mo2^viii^—Mo3—Mo2^x^	112.05 (2)	S2^xvi^—Na2—Mo1^xvii^	43.72 (2)
S3^vii^—Mo3—Mo2^ix^	59.911 (14)	S2^iv^—Na2—Mo1^xvii^	139.27 (11)
S3—Mo3—Mo2^ix^	117.957 (16)	Mo3^iv^—Na2—Mo1^xvii^	87.79 (5)
S2^ix^—Mo3—Mo2^ix^	56.42 (2)	S3^iv^—Na2—Mo1^xvii^	80.39 (5)
S2—Mo3—Mo2^ix^	146.91 (3)	Na2^v^—Na2—Mo1^xvii^	79.33 (6)
Mo3^viii^—Mo3—Mo2^ix^	89.333 (10)	Na2^vi^—Na2—Mo1^xvii^	87.34 (5)
Mo3^vii^—Mo3—Mo2^ix^	60.116 (12)	Mo1—Na2—Mo1^xvii^	157.78 (11)
Mo2^viii^—Mo3—Mo2^ix^	146.475 (18)	S5—Na1—S2^xviii^	100.69 (9)
Mo2^x^—Mo3—Mo2^ix^	57.956 (12)	S5—Na1—S2^iv^	100.69 (9)
S3^vii^—Mo3—Mo2	59.911 (14)	S2^xviii^—Na1—S2^iv^	116.64 (6)
S3—Mo3—Mo2	117.957 (16)	S5—Na1—S2^ii^	100.69 (9)
S2^ix^—Mo3—Mo2	146.91 (3)	S2^xviii^—Na1—S2^ii^	116.64 (6)
S2—Mo3—Mo2	56.42 (2)	S2^iv^—Na1—S2^ii^	116.64 (6)
Mo3^viii^—Mo3—Mo2	89.333 (10)	S5—Na1—S1^iv^	71.10 (8)
Mo3^vii^—Mo3—Mo2	60.116 (12)	S2^xviii^—Na1—S1^iv^	66.58 (2)
Mo2^viii^—Mo3—Mo2	57.956 (12)	S2^iv^—Na1—S1^iv^	65.67 (2)
Mo2^x^—Mo3—Mo2	146.475 (18)	S2^ii^—Na1—S1^iv^	171.77 (17)
Mo2^ix^—Mo3—Mo2	110.679 (19)	S5—Na1—S1^xviii^	71.10 (8)
S3^vii^—Mo3—Na2^iv^	117.20 (8)	S2^xviii^—Na1—S1^xviii^	65.67 (2)
S3—Mo3—Na2^iv^	69.11 (8)	S2^iv^—Na1—S1^xviii^	171.77 (17)
S2^ix^—Mo3—Na2^iv^	61.70 (3)	S2^ii^—Na1—S1^xviii^	66.58 (2)
S2—Mo3—Na2^iv^	61.70 (3)	S1^iv^—Na1—S1^xviii^	110.03 (8)
Mo3^viii^—Mo3—Na2^iv^	125.80 (8)	S5—Na1—S1^ii^	71.10 (8)
Mo3^vii^—Mo3—Na2^iv^	174.20 (8)	S2^xviii^—Na1—S1^ii^	171.77 (17)
Mo2^viii^—Mo3—Na2^iv^	93.43 (4)	S2^iv^—Na1—S1^ii^	66.58 (2)
Mo2^x^—Mo3—Na2^iv^	93.43 (4)	S2^ii^—Na1—S1^ii^	65.67 (2)
Mo2^ix^—Mo3—Na2^iv^	117.90 (3)	S1^iv^—Na1—S1^ii^	110.03 (8)
Mo2—Mo3—Na2^iv^	117.90 (3)	S1^xviii^—Na1—S1^ii^	110.03 (8)
Mo1—S1—Mo1^iii^	67.06 (3)		

Symmetry codes: (i) *x*+1, *y*, *z*; (ii) *x*−*y*, *x*−1, −*z*; (iii) *y*+1, −*x*+*y*+1, −*z*; (iv) −*x*+1, −*y*, −*z*; (v) −*x*+*y*+2, −*x*+1, *z*; (vi) −*y*+1, *x*−*y*−1, *z*; (vii) −*x*+*y*+1, −*x*, *z*; (viii) −*y*, *x*−*y*−1, *z*; (ix) *x*, *y*, −*z*+1/2; (x) −*y*, *x*−*y*−1, −*z*+1/2; (xi) *x*−*y*−1, *x*−1, −*z*; (xii) −*x*+*y*+1, −*x*+1, *z*; (xiii) *x*−1, *y*, *z*; (xiv) *y*+1, −*x*+*y*, −*z*; (xv) *x*+1, *y*, −*z*−1/2; (xvi) −*x*+1, −*y*, *z*−1/2; (xvii) *x*, *y*, −*z*−1/2; (xviii) *y*, −*x*+*y*, −*z*.
